# Influencing the adhesion properties and wettability of mucin protein films by variation of the environmental pH

**DOI:** 10.1038/s41598-018-28047-z

**Published:** 2018-06-25

**Authors:** Maria Sumarokova, Jagoba Iturri, Andreas Weber, Maria Maares, Claudia Keil, Hajo Haase, José Luis Toca-Herrera

**Affiliations:** 1 Institute for Biophysics, Department of NanoBiotechnology, BOKU University for Natural Resources and Life Sciences, Vienna, Austria; 2 Department of Food Chemistry and Toxicology, Technical University (TU) Berlin, Gustav-Meyer-Allee 25, D-13355 Berlin, Germany

## Abstract

Mucins, the main component of the mucus secretions of goblet and epithelial cells, are known for exhibiting a different behaviour in accordance with their surrounding environment (i.e. among others the environmental pH), which induces a drastic change in their measured mechanical properties. In this work, we have first employed Atomic Force Microscopy (AFM) in Force Spectroscopy mode to evaluate the adhesion of porcine mucin films at the nanoscale, and the changes caused in this particular factor by a pH variation between 7.0 and 4.0, both quite common values in biological conditions. Measurements also involved additional varying factors such as the indenting tip chemistry (hydrophobic vs hydrophilic), its residence time on the measured film (0, 1 and/or 2 seconds), and increasing pulling rates (ranging from 0.1 up to 10 µm/s). A second approach regarded the macroscale behaviour of the films, due to their potential applicability in the development of a new set of stimuli-responsive biomaterials. This was possible by means of complementary Wilhelmy plate method (to test the wetting properties) and cell proliferation studies on films previously exposed to the corresponding pH solution. According to our results, treatment with lowest pH (4.0) provides porcine mucin with a more hydrophilic character, showing a much stronger adhesion for analogous chemistries, as well as enhanced capability for cell attachment and proliferation, which opens new pathways for their future use and consideration as scaffold-forming material.

## Introduction

The extended presence of mucous secretions in nature, in very diverse bodies and types of applications, emphasizes the versatility and important functionality of these coatings. Through evolution, mucus has turned into a key factor towards performance of some relevant activities in different species. Attending to its consistency and composition the role of such layers can range from mere lubrication to others such as protection or anti-fouling, capture of foreign bodies, and even attachment fixation, when acting as gluing compound. The latter can be, for instance, observed in mussels which firmly attach to rocks by secretion of a proteinaceous layer, in which the amino acid derivative 4,5-dihydroxyphenylalanine (DOPA) is a major component, able to mediate adhesion underwater^1^. Another example related to the animal kingdom would be that of tree and torrent frogs, which complement their impressive hierarchical toe pad structure^2,3^ with the delivery of a mucous layer to provide additional stability. Even snails use similar adhesive secretions in combination with their muscular ventral-foot to even climb in more slippery conditions^4^. Within the human body, mucus coatings cover many different locations, including the inner linings of organs of the respiratory tract, the gastrointestinal tract, the reproductive tract and the ocular surface, among others^5^. Despite the various roles played, all those different mucus examples share in common that they act as primary interface with their surroundings. Such a barrier is responsible, for instance, for the trapping of drugs, nutrients, or ions, which turns critical for many of the processes those organs are designed for^6^. A good example of it could be the interaction of Zinc (Zn²^+^) with mucins in the intestinal tract^7,8^. Zn²^+^ is essential for the maintenance and development of immune cells of both the innate and adaptive immune system. Moreover, its deficiency affects immune cells, resulting in altered host defence, increased risk of inflammation, and even death^9^.

Mucins, in turn, are the main component of the mucus secretions of goblet and other epithelial cells. These glycoproteins constitute a family of macromolecules which exhibit chemical differences depending on the organ they are present at, although their basic structure remains very similar^10,11^. Despite such a little structural variation, and mostly because of the relevant role abovementioned, an optimal characterization of their mechanical properties in different environments (e.g. pH) might shed some light on their adaptation to the very harsh conditions they are exposed to^12–14^. For instance, changes in their adhesiveness might be significant enough to induce a malfunctioning, or to shift between lubricant and sticky states, or *vice versa*. Indeed, some studies on *ex-vivo* mucosal tissues already reported on the existing adhesive difference upon variations in the environmental conditions, ranging from intense adhesion peaks observed at low pH (=2) to a more attenuated adhesion when pH was increased to a value of 7.0^15^.

Over the last thirty years, Atomic Force Microscopy (AFM) has proven itself as a technique of high versatility. In addition to its capability to deliver sample imaging in high resolution, it also enables the use of the Force Spectroscopy mode, which allows mechanical testing of the sample of interest^16^. In these experiments, an AFM-tip (e.g. conical shape, pyramid), a colloidal probe^17^ or even a living cell^18^ is first extended towards and then retracted from the targeted material by exclusively following the Z-axis. Such motion takes place under controlled displacement speeds, as governed by the movement of the piezo. During this process, the deflection of the cantilever is determined as a function of the displacement of the piezo-scanner^19^, and the force sensed by the cantilever can be calculated using Hooke’s law (which is equal to the cantilever deflection times its spring constant). Attending to which segment of the thus generated force vs distance plot is analysed, information related to very diverse factors such as Young’s Modulus, stiffness, relaxation time, viscosity or adhesion of the material under analysis can be determined. In the particular case of adhesion, its value derives from the minimum in the segment depicting the retraction motion of the indenter from the surface, and it is quite extensively used as characterization parameter in materials science^20^.

In the current work, the adhesion of commercial porcine-derived mucin films was firstly tested at the nanoscale by means of Atomic Force Microscopy. The performance of the films was investigated after exposure to two different pH values (7.0 and 4.0). Measurements were also done under varying residence times (0, 1 and 2 s) and pulling rates (between 0.1 and 10 µm/s), two factors that have recently been shown to influence the adhesion yielded^21^. Complementarily, and in order to extract wettability-related information, the indentation was performed by either hydrophobic (untreated) or hydrophilic (plasma activated) silicon nitride tips. Extrapolation of the obtained wetting results to the macroscale behaviour of the films, for the sake of studying their potential applicability in the development of a new set of stimuli-responsive biomaterials, was possible by *Wilhelmy plate* method and cell proliferation studies on films previously treated with the corresponding pH solution. Hence, treatment with lowest pH values transforms porcine mucin into a more hydrophilic, stickier, and cell binding promoting material than its pH 7.0 treated analogous version, which opens its future possibilities as scaffold-forming material or as part of other biomedical devices, as happened to some of the natural examples mentioned above^22,23^.

## Results

### Adhesion force: interplay between mucin wetting and tip chemistry

**Figure 1:**
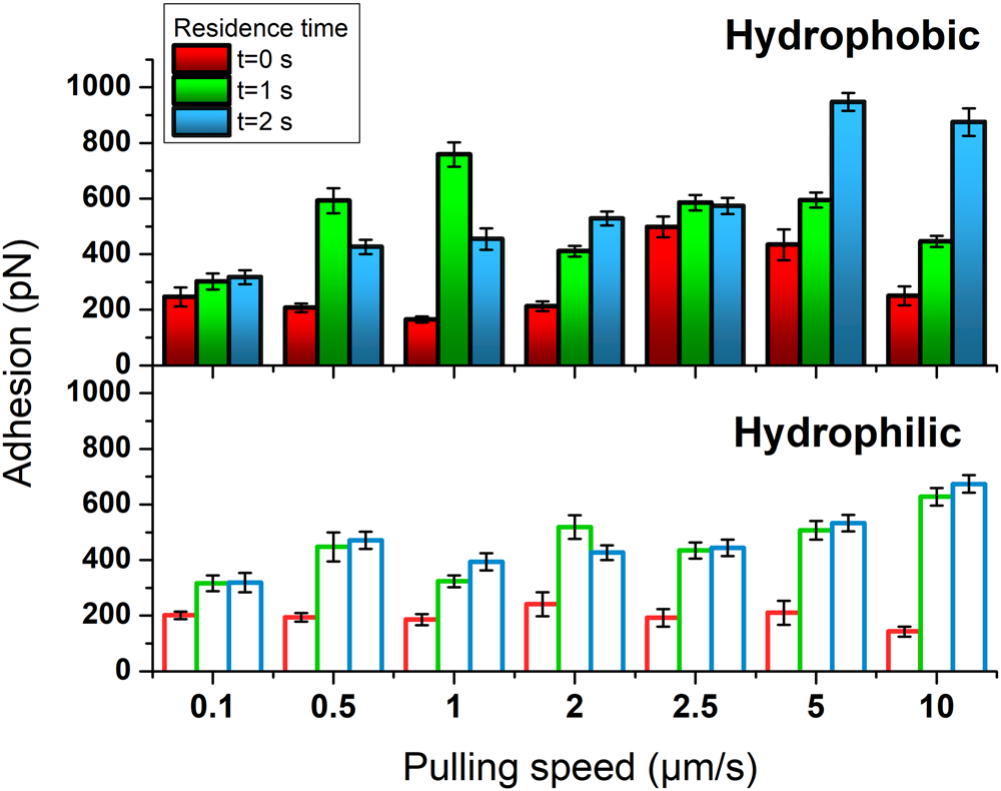
Adhesion force between a silicon nitride tip (hydrophobic –top- and hydrophilic –bottom-) and the mucin film at pH 7.0, under varying contact/residence (dwell) time and pulling rate. Error bars indicate the mean error for N > 50 plots.

The maximum adhesion/detachment force (interaction between the AFM tip and the film) was measured for mucin films on glass at pH = 7.0, for increasing pulling rates and two different residence times, as summarized in Fig. 1. A first trend can be rapidly noticed: The adhesion force showed a substantial increase with the pulling rate, which turned even more evident for longer dwell times. In particular, the magnitude of the measured adhesion remained higher along most of the pulling rates applied in those experiments where an untreated silicon nitride tip was employed. For a residence time of 0 seconds, meaning immediate retraction upon maximum load achievement, the pulling rate did only influence the adhesion between the AFM tip and mucin for untreated probes. Upon indentation with hydrophilic (plasma-activated) tips, the adhesion force hardly varied, staying in values below 250 pN, which represents around a half of the maximum values obtained under indentation with a more hydrophobic probe. These lower tip-mucin detachment forces for plasma-treated probes point towards a hydrophobic character of the mucin film in these conditions (pH 7). An exhaustive analysis over the significance of the measured variations can be found in the Supporting Information (see, *Appendix* section).

### Influence of the environmental conditions

**Figure 2:**
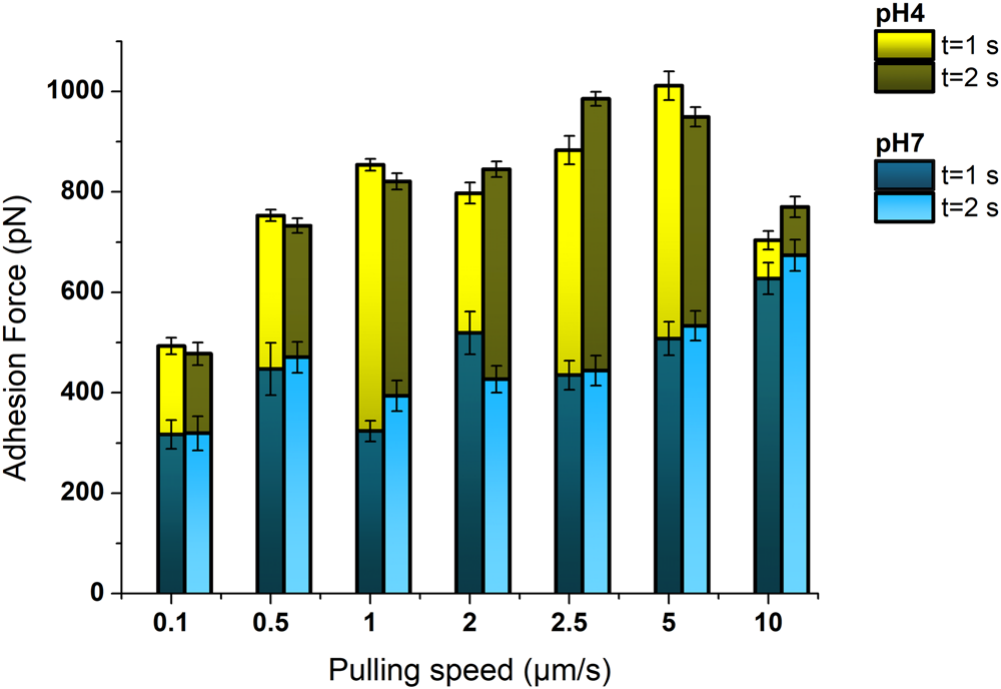
Adhesion force of mucin films as obtained from its contact with a hydrophilic tip for varying dwell times, and after exposure of the film to two different pH values: 4.0 – yellow- and 7.0 –blue-. Error bars indicate the mean error for N > 50 curves.

Knowing the fundamental role of mucous films in nature (transport, capture, lubrication or hydration) and the different -as well as harsh- environments in which these appear (saliva, respiratory, cervix, gastro-intestinal tract, etc)^24^ an important factor to consider is the environmental pH. The way in which these conditions affect the physicochemical properties of mucus at the nanoscale is something yet only sparsely investigated, but might play a decisive functional role. Even though the mucus *in vivo* contains a full bunch of macromolecules, studying the performance of its most abundant component, mucin, might lead to a better understanding of its nature and activity. Hence, the maximum adhesion or, in other words, the maximum detachment force required, was compared for mucin films after being exposed to buffers in 2 different pH values, 7.0 (as obtained above) and 4.0, under hydrophilic tip-exerted load, dwell times of 1 and 2 seconds, and different pulling rates. Figure 2 depicts the results obtained. Interestingly, the adhesion force for all the pulling rates attempted increased rather drastically. These range between only around a 15% (as measured for 10 µm/s tip retractions), up to more than 2-fold in the most extreme cases. The increase appears to be completely independent of the time that tip and sample remained in contact. As mentioned above, the significance of the measured variations can be found in the Supporting Information (*Appendix* section).

However, the most relevant outcome derives from the change in the adhesion behaviour itself, which indicates a much higher affinity of pH 4-treated mucin for hydrophilic tips. These findings suggests that mucin undergoes a shift in its wettability upon (short) exposure to low pH solutions.

### Work of adhesion

**Figure 3:**
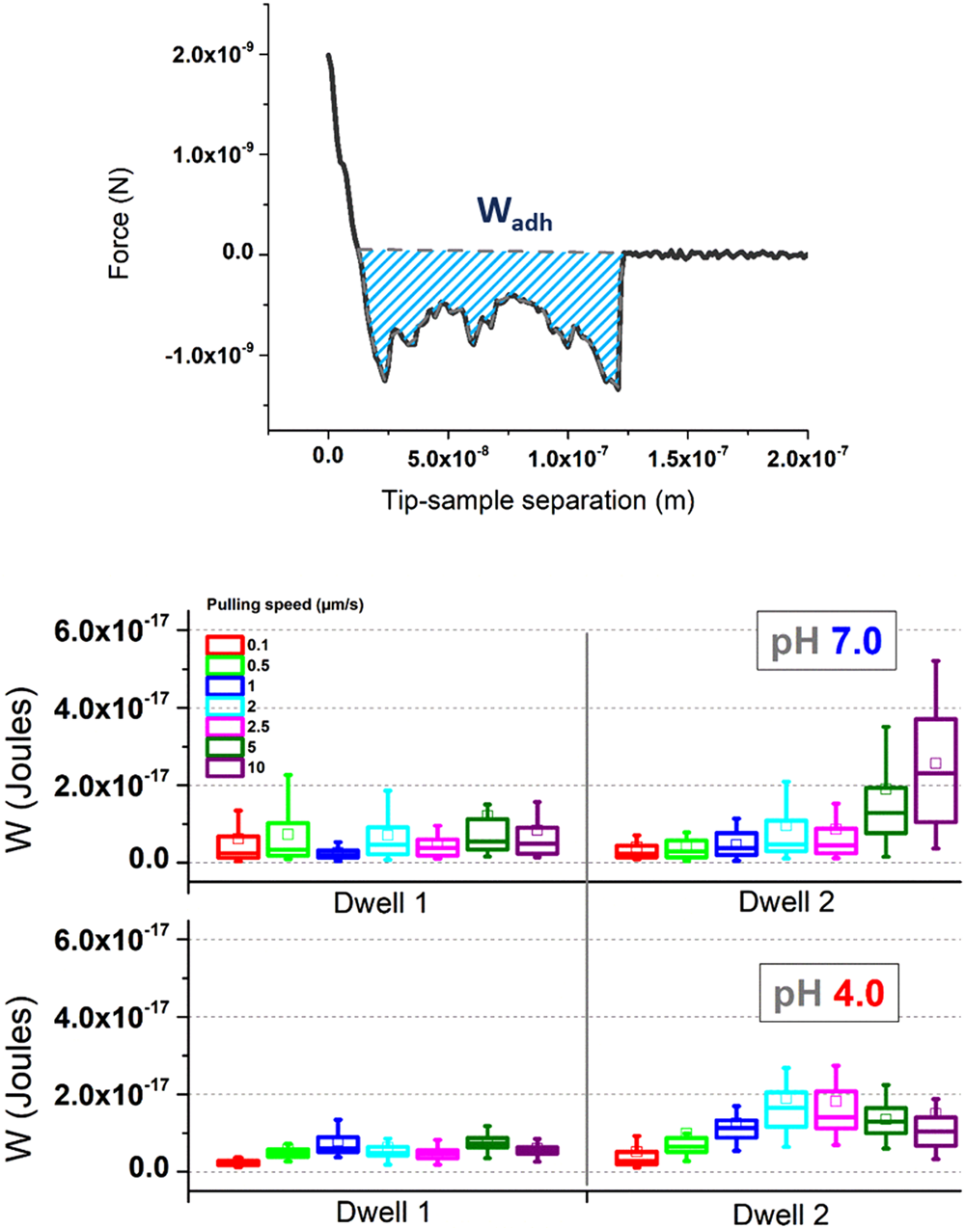
Work of adhesion. (Top) Representative retraction plot of mucin in pH 7. (Bottom) Chart showing the different work values of mucin under different pH, residence time and pulling speed. The square (□) in the box plot represents the mean value, the box-splitting horizontal line gives the median, and the upper and lower value are indicative of the achievement of either the 99% or 1% of the population, respectively.

Another factor to be taken into account is the so-called work of adhesion. This factor relates to the area measured under the retraction curve, due to adhesion-related events, before reaching back the zero force (Fig. 3-Top). Calculation of this parameter has been reported to become rather useful in systems involving cells^25^ or polymer coatings^26^, but it is widely used for the analysis of biomaterials in general.

The work of adhesion of mucin films was measured under five different experimental variables: Dwell times of 1 and 2 s, increasing pulling speeds, and two pH values (either 7.0 or 4.0) for the pre-treatment of the films. The resulting boxplot diagrams are collected in Fig. 3-Bottom.

Already at a first glimpse, these results allow discriminating between two different types of response. On one hand, the work required to break the tip-to-sample contact for pH 7-treated films appeared to be larger than for their pH 4-treated analogous samples under identical measuring conditions (residence time and retracting speed). Such results confirm the first observations reported for mucus-coated intestinal tissue^15^. In addition, a longer residence time (2 s) led to an increase in the measured values. This appears to be even more relevant under either more acidic treatment conditions or larger pulling speeds (for untreated silicon nitride tips). The explanation for such an increase after a pH 4 treatment derives from a broadening in the adhesion peak followed by a one-step rupture of the tip-sample contact, in comparison to the retraction plot observed after exposure to pH 7 (and shown in Fig. 3). Hence, the initial broadening of the adhesion peak seems to stay constant or even to induce a certain narrowing beyond some applied conditions, which compensates the simultaneous increase in adhesion force previously shown in Fig. 2, and keeps the energy values constant. This situation holds up to the extreme 10 µm/s pulling, where the tip-mucin contact rupture takes place in an abrupt manner under application of lower forces. Overall, values remained in the range between 2 * 10^−18^ and 2.5 * 10^−17 ^Joules (see complete set of values in TableSI1, Supporting Info) which agrees with results reported before for similar experiments^27^. The significance of the measured variations is found in the Supporting Information.

### Mucin macroscale properties variation with pH

**Figure 4:**
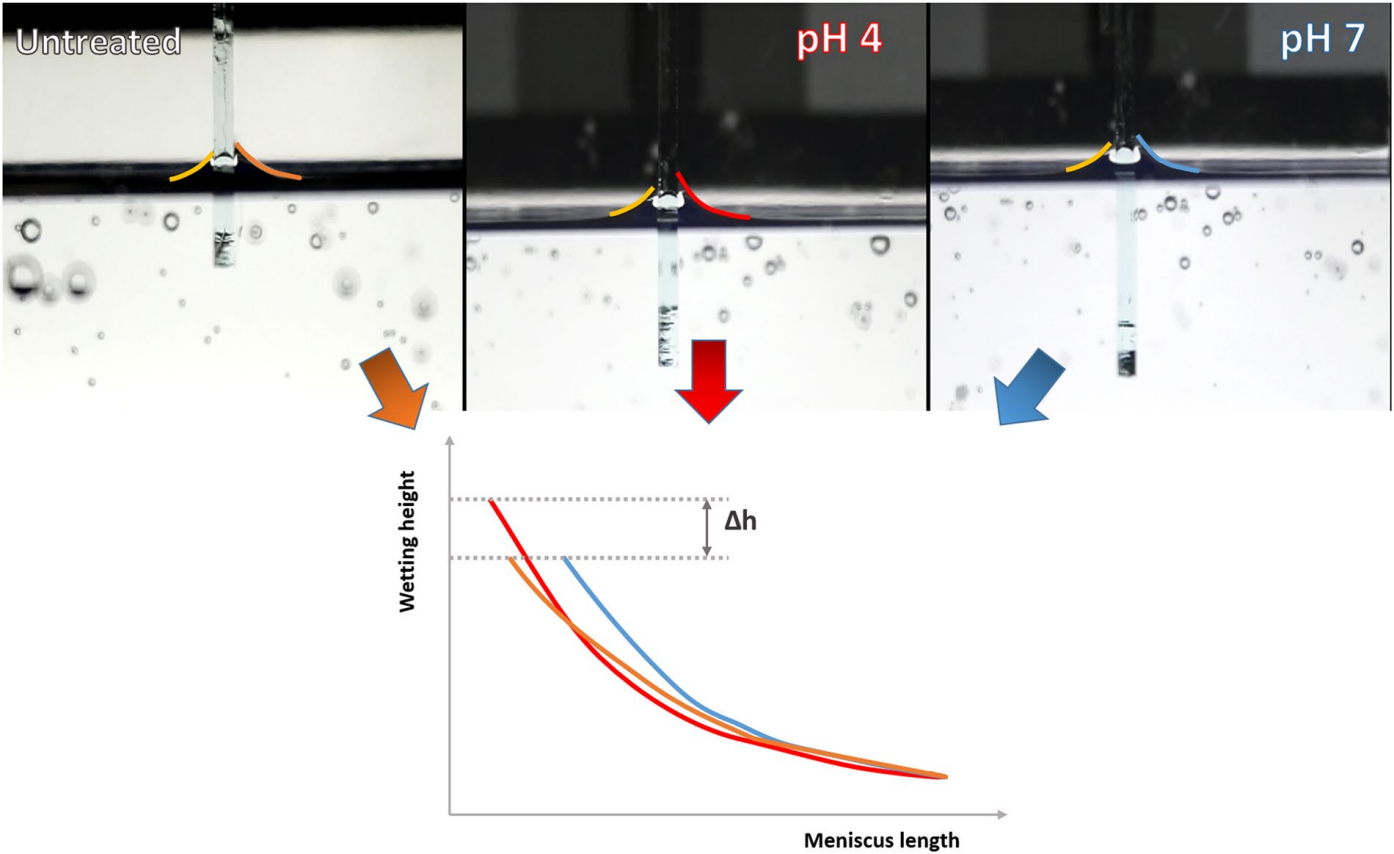
Wettability test performed following the Wilhelmy plate method. Coloured lines highlight the meniscus profiles resulting from immersion of the untreated (orange), pH 4 (red) and pH 7 (blue) mucin films in water. Left side of the glass slide remained uncoated.

Observations so far converged into an apparent wettability change of thin mucin films upon treatment with low pH buffers. In order to correlate these changes with their macroscopic performance, wettability of mucin films was tested by using the so-called Wilhelmy plate method (see *Materials and Methods* section). This technique allows testing the contact angle formed at the interface when mucin-coated glass slides in one of the three states under study (untreated, post-pH 4 or post-pH 7) are immersed in water. Comparison between experiments was only possible by employment of identical experimental conditions (e.g. immersing speed). Pictures showing the side view of a representative process are collected in Fig. 4 (additional experiments can be seen in Figure SI3, Supporting Information).

Overlapping of the resulting profiles clearly shows the existing difference between each of the mucin films: pH 4 treated coatings reach larger wetting heights (thus matching their increased hydrophilicity), compared to the other 2 systems, which show rather similar trends. Another difference to note derives from the variation of the meniscus curvature in the respective samples, leading to a capillary rise. The radius of such curvature inversely correlates to the pressure balance between water and the surrounding atmosphere (air), according to the Young-Laplace equation^28^. This factor, for instance, allows a subtle differentiation between untreated and post-pH 7 treatment films, which otherwise would be considered to be basically the same. The shape of the meniscus for untreated films shows an attenuated curvature as the distance to the surface decreases, suggesting a slight increase on its wetting properties.

From the varying heights that water reaches in the wetting of the samples, determination of *adhesion energy* at the liquid-solid boundary phase is possible, which describes the wetting process of those systems in a more detailed manner. In this case, and due to the perpendicular positioning of the sample, the adhesion energy mainly relates to a change in the potential energy (E_p_) of the liquid (meniscus). Such a change is brought by the mass of water that is able to climb, as occurs in the case of the pH 4 treated mucin film. Since this factor exclusively depends on the height, the pH 7-treated and untreated cases can be considered being the same. An approximate value of the water mass being involved is then calculated from the shape of the triangle formed (see Figure SI4), which corresponds to the volume of a half cone. This is described by the following equation (Equation 1):

1 $${V}_{cone}=(\pi {r}^{2}\ast h)/3$$

where *r* and *h* represent the radius (0.2 mm) and height (0.35 mm) of the triangle, respectively. This half-volume is then inserted in the potential energy equation (Equation 2), which in this particular case is defined as:

2 $${\rm{\Delta }}{E}_{p}=m\ast g\ast {\rm{\Delta }}h=\rho V\ast g\ast {\rm{\Delta }}h=\rho \ast \frac{Vcone}{2}\ast g\ast {\rm{\Delta }}h$$

where *ρ* is the density of water, *g* is the gravity acceleration and *∆h* represents the height variation. By substitution of the corresponding values and further adjustment of the units involved, the pH 4 treatment of mucin yields a difference of 2.514 * 10^−13^ J with the other two systems under study. This value is negative, since it corresponds to energy being released in the process of creation of a new liquid-solid boundary replacing the previous condensed phase-vapour boundaries.

### Mucin as a suitable substrate for cell culture

In order to test the impact of the wettability variations on the adhesion properties of those mucin films exposed to low pH values, cell proliferation studies were carried out. The cell lines employed were: HUVEC cells, a usual model system, and HT-29-MTX cells, a stable clone derived from the human colon adenocarcinoma cell line HT-29 treated with Methotrexate (MTX)^29^. These HT-29-MTX are capable of producing mucus, and thus mimic one of the natural states of mucins. For the experiments, pre-treated mucin samples were exposed to the corresponding cell line (72 h in the case of HT-29-MTX, and 24 h for HUVEC), and then measured by fluorescence microscopy upon staining with Calcein-AM.

**Figure 5:**
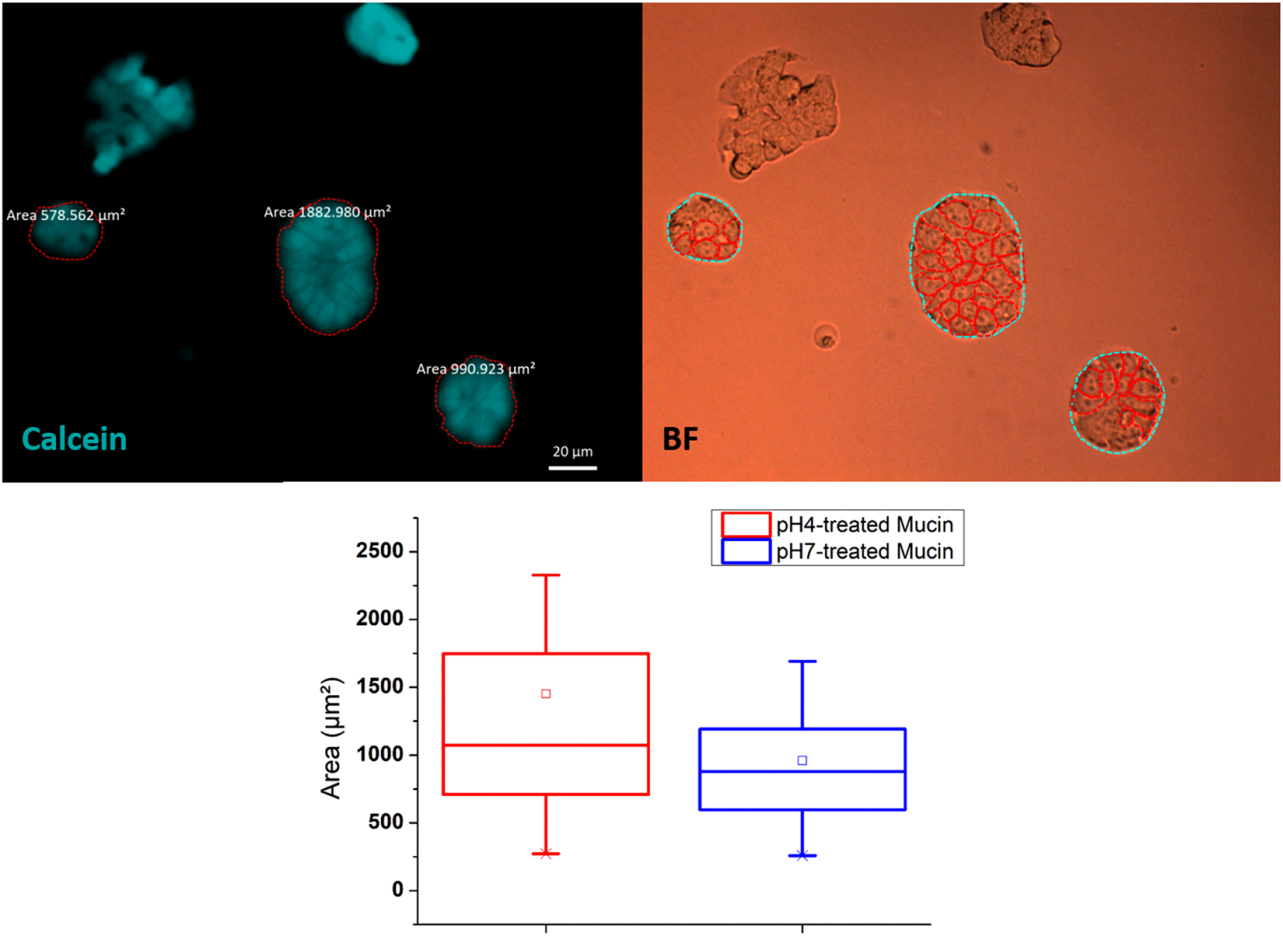
Fluorescence and brightfield optical micrographs of HT-29-MTX cells on mucin samples after 72 h. Red lines in bright field micrographs highlight the boundaries in between individual cells. Scale bar indicates 20 µm. Boxplot below shows the distribution of the area occupied by the cell aggregates on pH 4 (red) or pH 7 (blue) treated films. The square (□) in the box plot represents the mean value, the box-splitting horizontal line gives the median, and the upper and lower value are indicative of the achievement of either the 99% or 1% of the population, respectively.

HT-29-MTX cells showed a high tendency to aggregation in the shape of rather big circle-like structures, as shown in Fig. 5^30^. Although production of a noticeable external layer of mucus by these cells takes at least 96 h of incubation (data not shown), an incubation time of 24 h is sufficiently long as to enable a first appearance of membrane-anchored mucus points that may strengthen their binding towards the underlying mucin-coated substrates. In these conditions, the pH-treated mucin layers showed very distinctive behaviour in regard to the measured aggregate sizes. Hence, pH 4-treated mucin films favoured the formation of bigger tissue-like structures, with a mean value about twice the values observed for pH 7-treated films (1451 vs 959 µm²). Such a variation might be explained by the more suitable environmental conditions for HT-29-MTX development in the presence of a more hydrophilic substrate.

**Figure 6:**
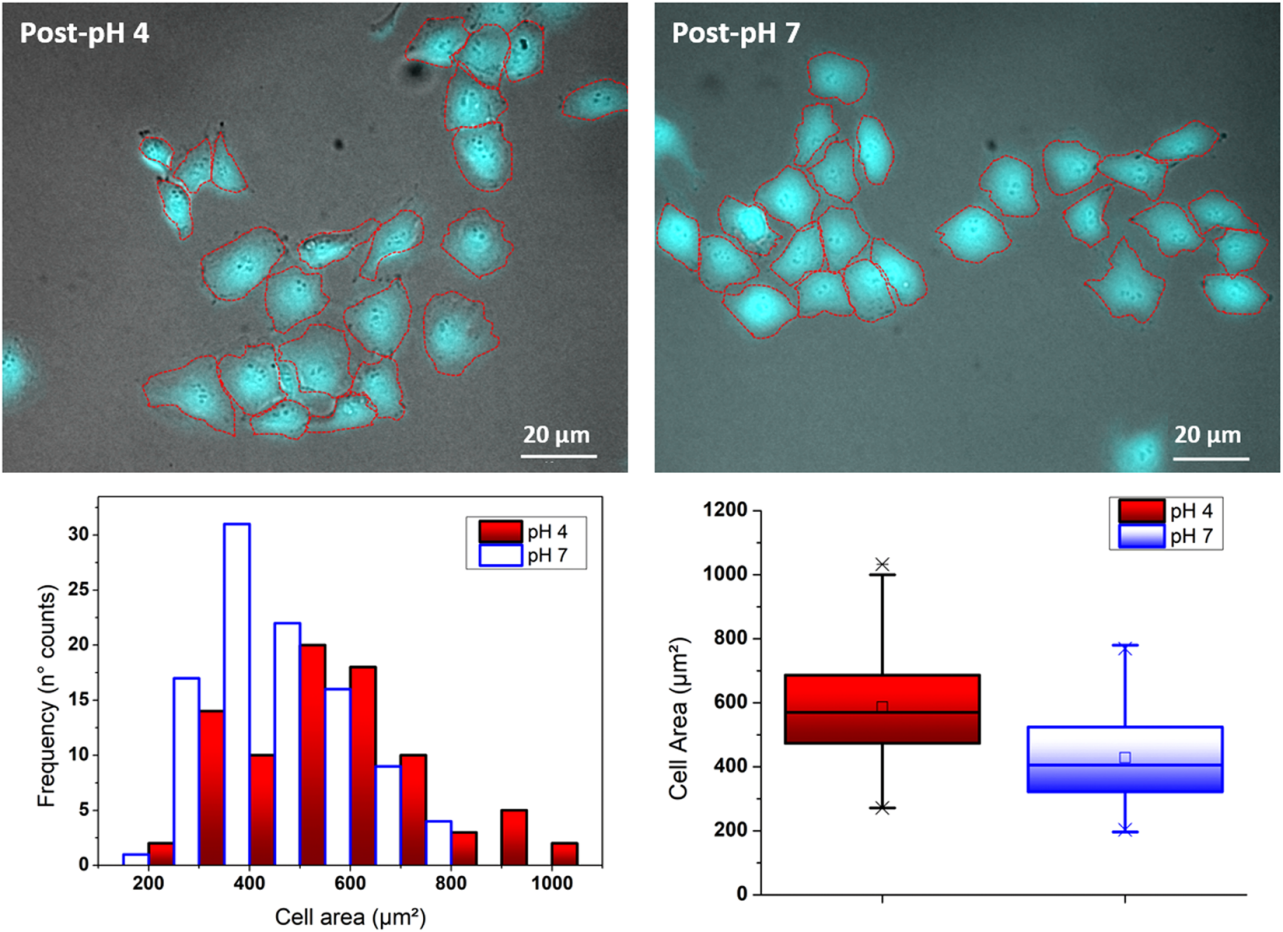
HUVEC cells on pH-treated mucin films after 24 h of incubation. Red lines highlight the cell profiles for a better viewing. Statistical analysis below show the individual cell area distribution in the shape of both histograms and boxplots. In the latter, the square (□) in the box plot represents the mean value, the box-splitting horizontal line gives the median, and the upper and lower value are indicative of the achievement of either the 99% or 1% of the population, respectively.

In turn, HUVEC cultured onto the more hydrophilic mucin (Fig. 6) appeared in a more spread state, with a mean body area of 586 µm², compared to the 428 µm² measured for the other set. The shifting in size distribution can be identified almost straightaway from the respective histogram plots.

## Discussion

The search and use of naturally obtained (bio)polymers in the design of new interfaces has given rise to a number of potential applications. Among these biopolymers, those smoothly adapting to biological conditions have become a group under close observation because of their candidacy for the design of diverse biomedical devices or cell scaffold materials. Mucin, for instance, is the major component in human body’s mucous secretions: lung, salivary and gastric mucins, etc^5^. Therefore, accessibility to its collection/extraction seems to be rather unlimited. A very interesting feature lies in the capability mucin shows to adapt to a large number of environments (some being quite extreme) without losing its functionality. Hence, it might act as lubricant or gluing agent, trap essential elements or inoculated drugs or become an effective protective layer, depending on both the environmental conditions and the corporal needs. Such a versatile behaviour turns mucin into a true potential component in terms of designing thin film with *on demand* tuneable properties.

By the aforementioned adaptation, mucin seems to undergo variations in its fluid state, mainly affecting to the measured viscosity of the material^12^. This viscosity shift implies a different stickiness or, in other words, modification of its adhesive properties. Since Atomic Force Microscopy has been proven to be a very efficient tool, when operated as a mechanical device, to characterize variations in those type of mechanics-related properties (i.e. stiffness, adhesion) for similar systems (polymers, gels, etc) and under similar stimuli (i.e. charge density-induced adhesion variations)^31–33^, its applicability to mucin films is considered as an optimal choice.

The current data provide strong evidence on how adhesion of mucin films depends on two factors: the pulling rate and the residence time. Keeping an accurate control over these two parameters is crucial in order to determine the response of the films. The longer a particle or a foreign body stays in contact with the mucin, the higher the stickiness observed. In this interaction the environmental pH has shown to be of extreme relevance. Exposure of the mucin film to a solution of pH 7.0 prior to the measurements, a pH which can be found in different parts of the human body, translates into a gain in the hydrophobic character of the film. Contrarily, its treatment with pH 4 buffer shifts the system wettability towards a more hydrophilic state. The work of Sortres *et al*.^15^, as aforementioned, already analysed the adhesion parameter for mucosal surfaces that were directly collected from the distal part of the small intestine of adult pigs. These samples showed to have very distinctive behaviour depending on the pH value. Hence, at pH 2.0 a very sharp adhesion peak followed by a sudden, one-step, recovery of the zero force was observed. Under pH 7 such recovery followed a completely different pathway, with a much longer mucin extension until contact rupture occurred. These results depict very nicely the change taking place, but, unfortunately, the measuring conditions employed by those authors (Probe diameters ≥ 15 µm, pulling speeds ca. 70 µm/s) made extrapolation to our particular case just impossible. A measuring system resembling better -in terms of scale- our current study is that of Popeski-Dimovski^27^. However, experiments in that work were performed following an opposite approach: hydrophilic tips were coated with mucins, and then employed to measure their binding affinity to alginate-based hydrogels. The author also fixed the pH value to 7.4, thus mimicking conditions in the small intestine, and tried increasing the tip-to-substrate interaction time, which resulted in a work of adhesion of around 3.6 * 10^−19^ J, for a dwell time of 5 s and under loads of only 200 pN (a 10% of the current load applied).

Such a variation of mucin properties, already important at the nanoscale, also affects the macroscopic performance of the film, as proven by Wilhelmy plate results (Fig. 4) and the complementary cell attachment studies (Figs 5 and 6). Both methodologies suggest a clear enhancement in the wetting capability of mucin after immersion in pH 4. It is of particular interest that such a treatment has proven to be effective in terms of generating cell appealing interfaces. Because of the long-term contact (at least far above the timescale here observed) of cells on top of mucin, which might strengthen the affinity between both, together with the interaction of the latter with ion/drugs/nutrients, the employment of this biopolymer as scaffold can be surely envisaged. Chemical modification of the administered drugs, attending to the wetting/stickiness state of the mucus layer in the particularly applied environmental conditions, can also play a critical role in their interaction and efficiency^34^.

## Materials and Methods

### Sample preparation

Borosilicate circular cover glasses (diameter: 24 mm, thickness: 0.08–0.12 mm, Menzel Gläser) were sonicated in ethanol, dried under N_2_, and cleaned by oxygen plasma (Gala Instrumente, Bad Schwalbach, Germany). After that every glass was immersed for about 1.5 h into the coating solution which contained 1 mg/mL of commercial porcine mucin in phosphate buffered saline buffer (PBS, pH = 5.5). After rinsing in ultrapure MilliQ water (pH 5.5, resistivity 18.2 MΩ cm), the samples were stored at 4 °C, immersed in water, until usage. Samples were rinsed with water before placing them on the AFM stage. For pH-treated samples, these were incubated for 1 h in the corresponding buffer (Citrate or PBS), and again washed with water prior to being measured. An additional step consisting on drying by N_2_ was also performed before wetting experiments by Wilhelmy method.

### Cell culture

Human umbilical vein endothelial cells (HUVEC) employed in this work were kindly provided by Dr. Spela Zemljic-Jokhadar (Medical Faculty, University of Ljubljana). In turn, goblet cell line HT-29-MTX-E12^29^ were obtained from the European Collection of Cell Cultures (ECACC, Porton Down, UK). Cells were cultivated in DMEM GlutaMAX supplemented with 10% Fetal Bovine Serum (FBS) and 1% penicillin/streptomycin at 37 °C and 5% CO_2_. For fluorescent staining, cells (4 × 10^4^) were seeded on pre-functionalized glass slides and incubated for either 24 (HUVEC) or 72 hours (HT-29-MTX) at 37 °C. After washing with 1 × PBS, cells were incubated with Calcein-AM solution (1:100 dilution in PBS, stock: 1 mg/ml in DMSO) directly in the medium for 30 min at 37 °C, followed by three washing steps with 1x PBS. Then, Leibovitz’s L-15 medium was added and cells were directly imaged by fluorescence microscopy (Zeiss Axio Observer Z1, Carl Zeiss GmbH, Germany).

### Film wettability test

Sample wettability tests were performed according to the so-called *Wilhelmy Plate* method^28^, which allows determining the advancing contact angle as a solid sample is immersed in a liquid. In our case, measurements regarded the use of mucin coated microscope glass covers before and after pH treatment, and ultrapure MilliQ water as bulk liquid. Uncoated glass covers were also employed as reference. Mucin film samples were prepared following the protocol described above. Motion of the slides was controlled with a D-3400 dip-coater (Mayer Feintechnik GmbH, Göttingen, Germany). Collected side view pictures and videos (see Supporting Information) were recorded with a 13 MP camera (OIS, RGBW, AF, dual-tone) mounted in a Huawei CRR-L09 cell phone, in combination with a LED light panel (Kaiser Fototechnik, Buchen, Germany) to improve the brightness and contrast.

### Quartz crystal microbalance with dissipation (QCM-D)

QCM-D experiments were performed in a Q-Sense E4 instrument (Q-Sense AB, Gothenburg, Sweden). Prior to their use, SiO_2_ coated quartz sensors were sonicated in ethanol, dried under N_2_, pre-activated with UV/ozone (BioForce Nanosciences, Ames, IA, USA) for 30 min, and then mounted into the QCM-D chamber.

Variations in frequency (∆f) and dissipation (∆D) parameters were measured for mucin film formation at 25 °C (Supporting Info, Figure SI1). Data were collected from several overtones (n = 3, 5, 7,…,13) throughout the QCM-D experiment. In this work, we present the results from overtone 3. Complementarily, ∆D vs ∆f plots (Df plots) represent the simultaneous dissipation and frequency changes at a certain time, which provides a more detailed view on the viscoelastic evolution of films per mass unit (∆m) change.

### Scanning Electron Microscopy (SEM)

Topography of the coated glass slides could be also visualized by SEM (FEI Inspect S50 SEM, USA), as shown at the Supporting Information section (Figure SI2). Prior to their measurement, samples were fixed with glutardialdehyde (0.25% in 100 mM phosphate buffer, pH 7.2) for 20 min, which avoided drying aggregation effects, thoroughly rinsed in water, dried at 40° and subsequently gold sputtered.

### Atomic force microscopy (AFM)

AFM measurements were performed at room temperature (23 °C) by using a Nanowizard I (JPK Instruments, Berlin, Germany) in force spectroscopy mode. Silicon nitride cantilevers DNP-S10 (Bruker, USA) were used in all experiments. The cantilevers were calibrated before experiments using the thermal noise option of the JPK software. The spring constant of the cantilevers was around 0.12 N/m. The so-defined “hydrophobic” or untreated tips went through a short cleaning step prior to their use, to avoid potential contribution from contaminants, which consisted of a dipping in ethanol and a gentle drying with N_2_. In turn, achievement of “hydrophilic” tips involved the exposure to oxygen plasma for 30 s. Force spectroscopy measurements were carried out in order to evaluate the adhesion forces of mucin. Two experimental parameters were taken into account: first, the loading rate (approaching and retracting speed of the cantilever) and second, the residence time of the cantilever on the sample (also called dwell time in the text). In particular, the approaching-retracting speeds were 0.1, 0.5, 1, 2, 2.5, 5, and 10 µm s^−1^; while three residence times were taken into account: 0, 1, and 2 s. One hundred force-distance curves were collected for every case for, at least, three different samples. The maximum force applied was 2 nN. Control experiments were carried out on borosilicate glass.

In addition, AFM imaging of the prepared film was performed in tapping mode with a JV-scanner controlled by NanoScope V controller (Bruker, USA) at scan rates lower than 2 Hz, and using Silicon nitride DNP-S10 probes (nominal spring constant, k = 0.12). Amplitude-setpoint values were optimized to prevent sample damaging. Prior to measuring, the system was left to equilibrate until the deflection signal was stable. All measurements were carried out in PBS. A representative micrograph is shown in Figure SI2.

### Data analysis

AFM images were treated with the Nanoscope software (Bruker, USA) and ImageJ. The data were plotted with Origin Lab 8.5. A minimum of 200 curves per loading rate and Dwell time were collected for the study. Fluorescence images were treated by Zen Blue Edition software, which also allowed determination of the cell body area as well as quantification of cells onto mucin films.

### Statistical analysis

Significance of the measured variations in adhesion force and adhesion energy (or work of adhesion) upon changing certain factors (pH, pulling speed, dwell time) was studied by means of statistical tools in Origin Lab 8.5. The resulting comparisons appear as *Appendix* section at the Supporting Information.

## Electronic supplementary material

Supporting Information

Wilhelmy Plate_pH 4-treated Mucin
