# Supplementary material for: Influencing the adhesion properties and wettability of mucin protein films by variation of the environmental pH
Source: Sci Rep. 2018 Jun 25;8:9660. doi: 10.1038/s41598-018-28047-z (PMC6018421; doi:10.1038/s41598-018-28047-z)
Supplement: Supplementary file 1 — Supporting Information [file 41598_2018_28047_MOESM1_ESM.docx]

**Influencing the adhesion properties and wettability of mucin protein films by variation of the environmental pH**

Maria Sumarokova^(1),§^, Jagoba Iturri^(1),§,^*, Andreas Weber^(1)^, Maria Maares^(2)^, Claudia Keil^(2)^, Hajo Haase^(2)^, José Luis Toca-Herrera^(1),^*


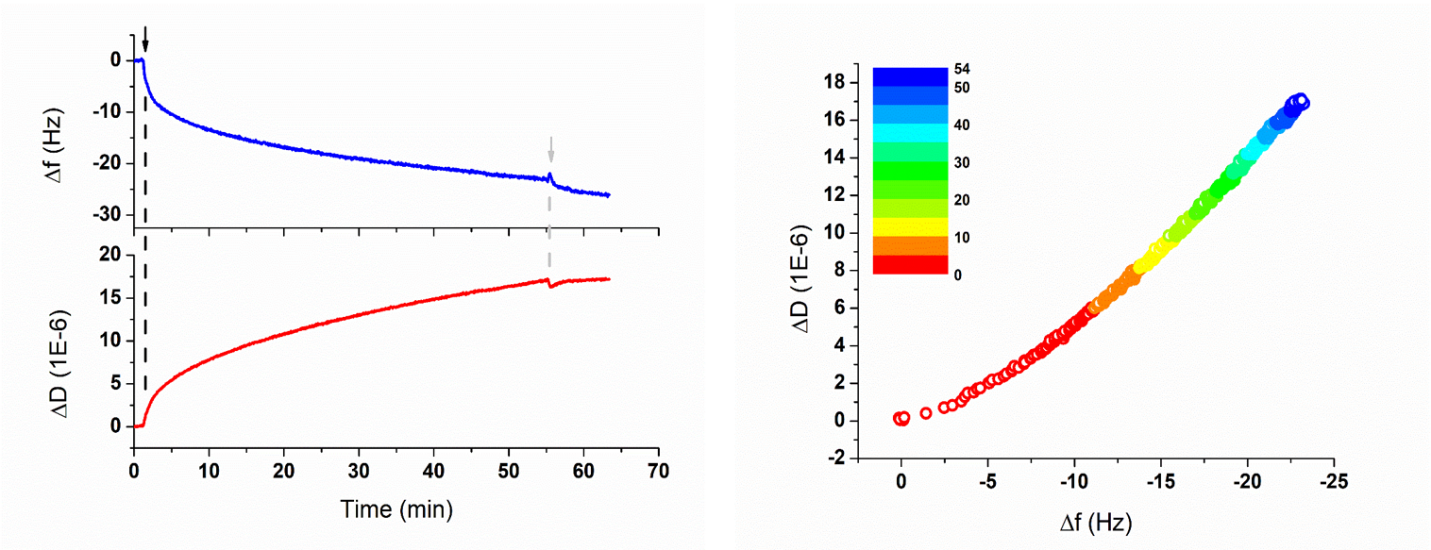


Figure SI1. Real-time mucin film formation on SiO_2_ as monitored by means of QCM-D. (Left) Frequency and Dissipation factor variation. Black and grey arrows indicate injection of mucin and of rinsing buffer, respectively. (Right) Df plot of the process. Colour scale indicates the time elapsed after injection of mucin protein.


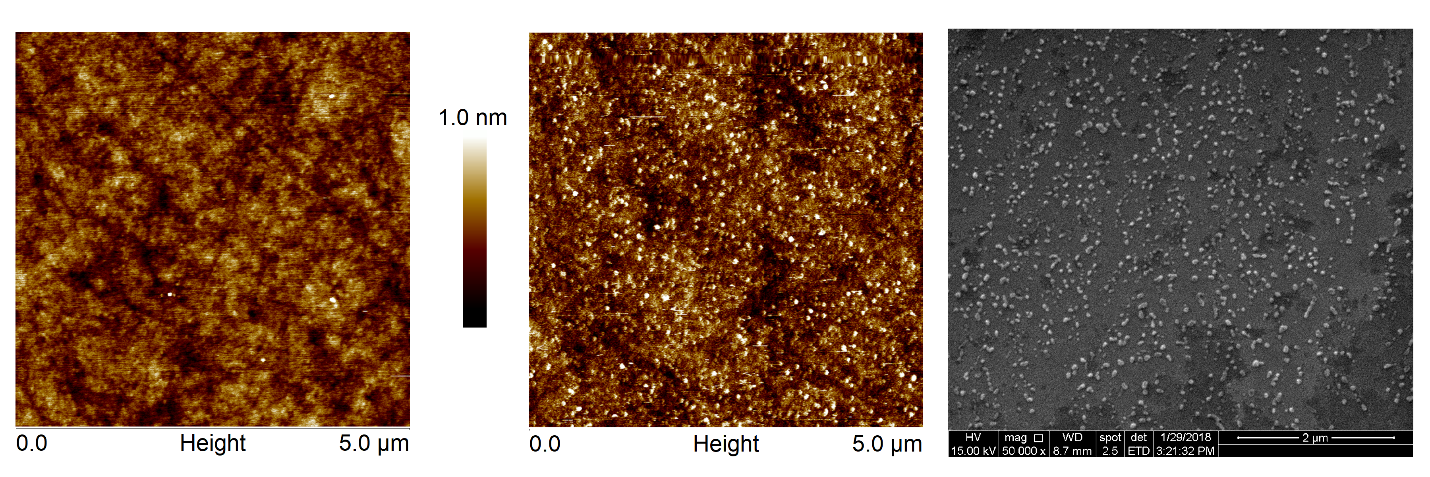


Figure SI2. Mucin film topography studies. Height micrographs from a clean SiO2 wafer (left) and Mucin film (center) as measured in tapping mode, on a 5 x 5 µm area. (Right) Scanning Electron Micrograph over the same measuring scale.


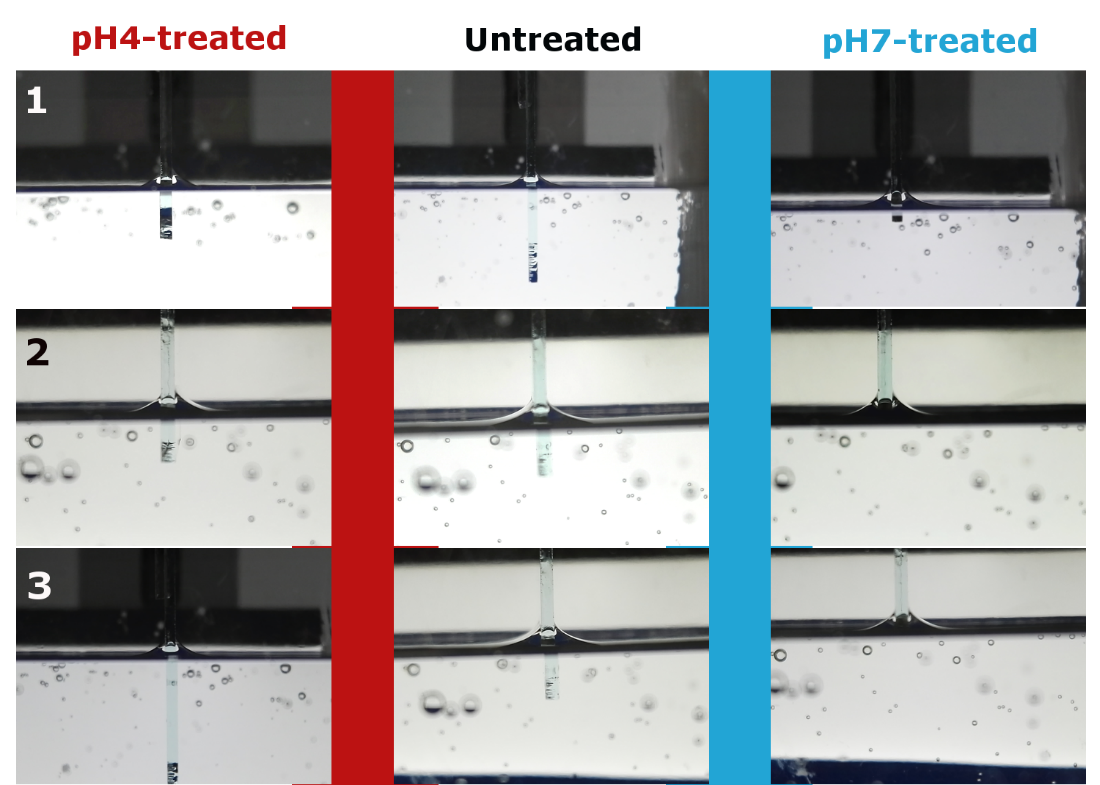


Figure SI3. Wilhelmy plate method image compilation. The columns show the profiles measured after treatment with pH4 (left) or pH (right), compared to untreated mucin films takes as reference (centre).


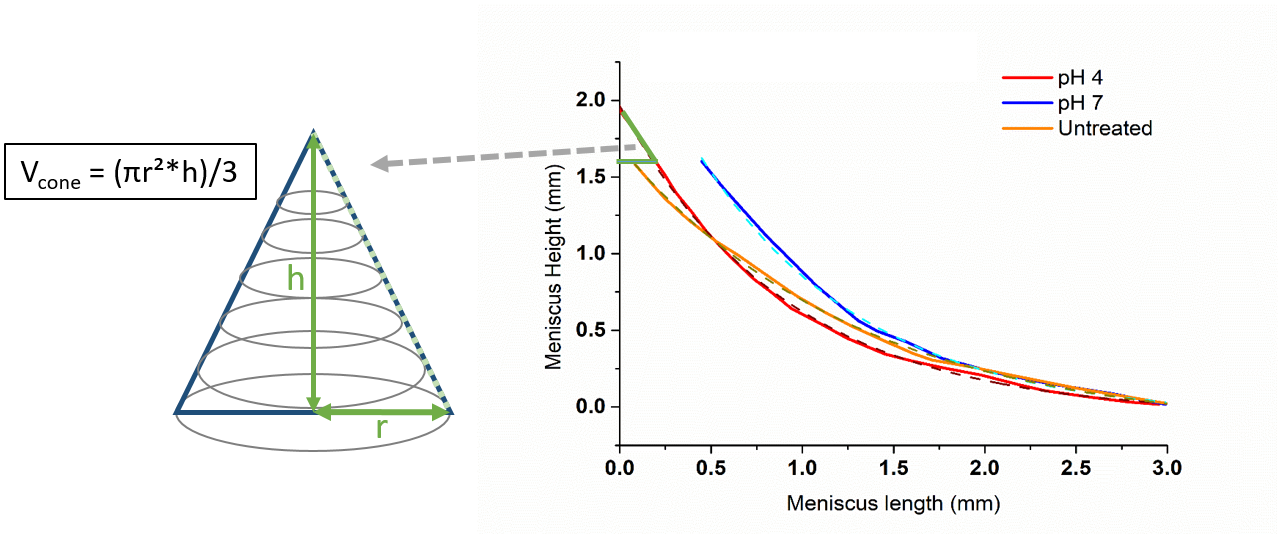


Figure SI4. Determination of the water volume derived from the difference in height between the meniscus profiles for potential energy calculation. The figure on the left highlights the half-cone fitting our particular case.

Table SI1. Work of adhesion values obtained when retracting a hydrophilic tip from a mucin film previously exposed to either pH4 or pH7 solutions. Measurements covered two different residence times.SE denotes the standard error of the mean value for a common total number of measurements N = 30

|  | **pH 4** | | | | **pH 7** | | | |
| --- | --- | --- | --- | --- | --- | --- | --- | --- |
|  | **Dwell 1** | | **Dwell 2** | | **Dwell 1** | | **Dwell 2** | |
| **Pulling rate** (µm/s) | W (J)  *10^-18^ | SE | W (J)  *10^-18^ | SE | W (J)  *10^-18^ | SE | W (J)  *10^-18^ | SE |
| **0.1** | 2.52 | 0.237 | 5.17 | 1.27 | 6.19 | 2.37 | 4.03 | 0.832 |
| **0.5** | 5.74 | 0.684 | 9.92 | 2.92 | 7.32 | 1.74 | 4.44 | 1.02 |
| **1** | 7.85 | 1.04 | 12.4 | 1.68 | 2.56 | 0.35 | 4.66 | 0.559 |
| **2** | 6.23 | 0.95 | 18.9 | 2.48 | 7.05 | 1.24 | 9.58 | 2.49 |
| **2.5** | 4.54 | 0.28 | 18.2 | 2.72 | 4.63 | 0.6 | 8.68 | 2.45 |
| **5** | 7.40 | 0.473 | 13.6 | 0.996 | 12.2 | 3.32 | 19.0 | 3.45 |
| **10** | 5.97 | 0.333 | 15.0 | 3.11 | 8.37 | 1.81 | 25.6 | 2.87 |

**Video file (additional attachment)**: [SI_Wilhelmy plate_pH4 mucin.avi](file:///\\Serverh80000\datah80000\h80000\H800\BIOPHYS\Jagoba\Manuscripts\2018%20-%20Sumarokova%20-%20pH-dependent%20adhesive%20properties%20of%20Mucin\Revision\SI_Wilhelmy%20plate_pH4%20mucin.avi)

**Appendix** - Statistical analysis for Adhesion plots

In this study, four different levels of significance (p> 0.05, p < 0.05, p < 0.01, p < 0.001) were defined. The colour scale shown will be maintained all over the results

| p > 0.05  (non-significant) | p < 0.05 (*) | p < 0.01 (**) | p < 0.001 (***) |
| --- | --- | --- | --- |

**Figure 1 – F_adh_ variation with pulling speed. Measurements at pH 7. Hydrophilic vs hydrophobic tip**

**a. Hydrophilic tip**

- **Normality testing.** (Mathematically using Shapiro-Wilk with 0.05, graphically using normal QQ-plots)

1. **t_0_:** Mathematically, only 2.5 µm/s is normally distributed. Graphically, all distributions are roughly normally distributed around mean value. (Exception 5 µm/s, has quite some outliers). Therefore, two sample **t-test fits to evaluate** data.
2. **t_1_:** Mathematically, no distribution is normally distributed. Graphically, all but 0.5 µm/s are sufficiently normally distributed around mean value. Therefore, **two sample t-test fit to evaluate** the data.
3. **t_2_:** Mathematically, no distribution is normally distributed. Graphically, all but 0.5 µm/s are sufficiently normally distributed around mean value (slow speed values have skew to left). Therefore, **two sample t-test fit to evaluate** the data.

- **Calculation of mean and standard error.**

|  | **Mean F_adh_ [pN]** | | |
| --- | --- | --- | --- |
| **Pulling speed [µm/s]** | t_0_ | t_1_ | t_2_ |
| 0.1 | 201.2 ± 13.3 | 316.8 ± 28.7 | 319.1 ± 34.2 |
| 0.5 | 193.9 ± 15.5 | 447.36 ± 51.9 | 470.6 ± 30.9 |
| 1 | 185.9 ± 20.2 | 323.6 ± 20.9 | 393.7 ± 30.6 |
| 2 | 240.7 ± 43.3 | 519.2 ± 42.3 | 426.9 ± 26.4 |
| 2.5 | 192.0 ± 31.7 | 434.9 ± 29.0 | 444.0 ± 29.8 |
| 5 | 210.4 ± 43.2 | 507.7 ± 33.4 | 533.3 ± 29.6 |
| 10 | 143.1 ± 18.1 | 627.7 ± 31.3 | 674.0 ± 31.2 |

**1. Differences of F_adh_ for the three dwell times t_0_, t_1_ and t_2_ at different pulling rates**

Here, variation of the adhesion data is compared for the different pulling rates (e.g. 0.1 µm/s compared to 0.5, 2 compared to 2.5 …) within a same residence time. Alpha = 0.05

*Table 1 Significance of F_adh_ change for increasing pulling speed and constant residence time*

|  | **p-value** | | |
| --- | --- | --- | --- |
| **Pulling speed [µm/s]** | t_0_ | t_1_ | t_2_ |
| 0.1 |  |  |  |
| 0.5 | 0.72263 | 0.02592 ^(*)^ | 0.00167^(**)^ |
| 1 | 0.75474 | 0.01955 ^(*)^ | 0.08033 |
| 2 | 0.19505 | 2.2864E-5^(***)^ | 0.41093 |
| 2.5 | 0.45576 | 0.09536 | 0.66845 |
| 5 | 0.73873 | 0.10111 | 0.03659 |
| 10 | 0.12661 | 0.00946^(**)^ | 0.00135^(**)^ |

**2. Differences of Fadh between dwell times for each pulling rates**

|  | **p-value** | | | |
| --- | --- | --- | --- | --- |
| **Pulling speed [µm/s]** | t_0_ | t_1 vs_ t_0_ | t_2 vs_ t_1_ |  |
| 0.1 |  | 5.874E-4^(***)^ | 0.95739 |  |
| 0.5 |  | 2.209E-4^(***)^ | 0.68829 |  |
| 1 |  | 4.279E-5^(***)^ | 0.05595 |  |
| 2 |  | 0.00177^(**)^ | 0.05893 |  |
| 2.5 |  | 0.00583^(**)^ | 0.82861 |  |
| 5 |  | 0.00209^(**)^ | 0.5667 |  |
| 10 |  | 7.194E-9^(***)^ | 0.29621 |  |

+ Comparison between t_0_ and t_2_

|  | **p-value** | |
| --- | --- | --- |
| **Pulling speed [µm/s]** | t_0_ | t_2 vs_ t_0_ |
| 0.1 |  | 7.662E-4^(***)^ |
| 0.5 |  | 6.163E-9^(***)^ |
| 1 |  | 4.413E-6^(***)^ |
| 2 |  | 0.00233^(**)^ |
| 2.5 |  | 0.00432^(**)^ |
| 5 |  | 5.893E-4^(***)^ |
| 10 |  | 1.418E-8^(***)^ |

**b. Hydrophobic tip**

- **Normality testing.** (Mathematically using Shapiro-Wilk with 0.05, graphically using normal QQ-plots)

1. **t_0_:** Normally distributed.
2. **t_1_:** Same.
3. **t_2_:** Same.

|  | **Mean F_adh_ [pN]** | | |
| --- | --- | --- | --- |
| **Pulling speed [µm/s]** | t_0_ | t_1_ | t_2_ |
| 0.1 | 212.8 ± 30.0 | 283.2 ± 28.4 | 313.8 ± 28.2 |
| 0.5 | 230.0 ± 13.7 | 641.2 ± 49.2 | 441.2 ± 30.5 |
| 1 | 188.1 ± 13.6 | 752.7 ± 43.5 | 499.2 ± 47.9 |
| 2 | 214.2 ± 16.5 | 441.5 ± 21.2 | 539.8 ± 25.6 |
| 2.5 | 387.0 ± 28.7 | 551.3 ± 24.9 | 591.2 ± 27.9 |
| 5 | 335.6 ± 43.1 | 614.1 ± 26.7 | 991.9 ± 32.2 |
| 10 | 256.9 ± 32.9 | 486.1 ± 20.6 | 885.9 ± 44.9 |

**1. Impact of increasing the pulling speed (constant residence time)**

|  | **p-value** | | |
| --- | --- | --- | --- |
| **Pulling speed [µm/s]** | t_0_ | t_1_ | t_2_ |
| 0.1 |  |  |  |
| 0.5 | 0.5515 | 3.364E-7^(***)^ | 0.00756^(**)^ |
| 1 | 0.03276^(*)^ | 0.09124 | 0.28598 |
| 2 | 0.2257 | 8.66E-12^(***)^ | 0.41884 |
| 2.5 | 8.396E-6^(***)^ | 9.142E-4^(***)^ | 0.18899 |
| 5 | 0.35248 | 0.08786 | 1.753E-18^(***)^ |
| 10 | 0.16132 | 1.863E-4^(***)^ | 0.04969^(*)^ |

**2. Comparison between different dwell times (constant pulling speed)**

|  | **p-value** | | | |
| --- | --- | --- | --- | --- |
| **Pulling speed [µm/s]** | t_0_ | t_1 vs_ t_0_ | t_2 vs_ t_1_ |  |
| 0.1 |  | 0.1213 | 0.45021 |  |
| 0.5 |  | 1.798E-11^(***)^ | 3.581E-4^(***)^ |  |
| 1 |  | 2.37E-19^(***)^ | 1.404E-4^(***)^ |  |
| 2 |  | 1.602E-11^(***)^ | 0.00324^(**)^ |  |
| 2.5 |  | 1.358E-4^(***)^ | 0.29493 |  |
| 5 |  | 2.061E-4^(***)^ | 2.002E-16^(***)^ |  |
| 10 |  | 1.473E-4^(***)^ | 8.572E-14^(***)^ |  |
|  | | |  |  |
|  | | |  |  |
|  | **p-value** | |  |  |
| **Pulling speed [µm/s]** | t_0_ | t_2 vs_ t_0_ |  |  |
| 0.1 |  | 0.03521^(**)^ |  |  |
| 0.5 |  | 1.246E-9^(***)^ |  |  |
| 1 |  | 2.893E-7^(***)^ |  |  |
| 2 |  | 1.602E-11^(***)^ |  |  |
| 2.5 |  | 5.124E-5^(***)^ |  |  |
| 5 |  | 7.555E-10^(***)^ |  |  |
| 10 |  | 5.697E-6^(***)^ |  |  |

**Figure 2 – F_adh_ vs pulling speed for hydrophilic tip, comparison of pH 4 and 7 at dwell of 1 and 2 s**

**Normality testing for pH 4.** (Mathematically using Shapiro-Wilk with 0.05, graphically using normal QQ-plots) **t_1_:** Mathematically, normally distributed. Can use two sample t-test for evaluation.

|  | Mean F_adh (Dwell t = 1s)_ | |
| --- | --- | --- |
| Pull speed [µm/s] | pH 4 | pH 7 |
| 0.1 | 493.1 ± 16.8 | 316.8 ± 28.7 |
| 0.5 | 753.2 ± 11.3 | 447.36 ± 51.9 |
| 1 | 854.2 ± 11.9 | 323.6 ± 20.9 |
| 2 | 797.6 ± 21.0 | 519.2 ± 42.3 |
| 2.5 | 883.1 ± 28.4 | 434.9 ± 29.0 |
| 5 | 1011.5 ± 28.5 | 507.7 ± 33.4 |
| 10 | 703.6 ± 18.3 | 627.7 ± 31.3 |

**t_2_:** Mathematically, normally distributed. Can use two sample t-test for evaluation.

**1. Differences of F_adh_ at t = 1 s between pH 4 and pH7 (same pulling speeds)**

|  | **p-value** | |
| --- | --- | --- |
| Pull speed [µm/s] | pH 4 | pH 7 vs pH 4 |
| 0.1 |  | 2.462E-6^(***)^ |
| 0.5 |  | 2.727E-9^(***)^ |
| 1 |  | 8E-55^(***)^ |
| 2 |  | 3.811E-9^(***)^ |
| 2.5 |  | 2E-22^(***)^ |
| 5 |  | 1E-23^(***)^ |
| 10 |  | 0.03617^(**)^ |

**2. Differences of F_adh_ at t = 2 s between pH 4 and pH7 treated samples (for same pulling speed)**

|  | Mean F_adh (Dwell t = 2s)_ | |
| --- | --- | --- |
| Pull speed [µm/s] | pH 4 | pH 7 |
| 0.1 | 477.6 ± 22.7 | 319.1 ± 34.2 |
| 0.5 | 732.7 ± 14.8 | 470.6 ± 30.9 |
| 1 | 821.0 ± 16.3 | 393.7 ± 30.6 |
| 2 | 845.2 ± 15.6 | 426.9 ± 26.4 |
| 2.5 | 985.4 ± 14.0 | 444.0 ± 29.8 |
| 5 | 949.4 ± 19.6 | 533.3 ± 29.6 |
| 10 | 770.2 ± 20.8 | 674.0 ± 31.2 |

|  | **p-value** | |
| --- | --- | --- |
| Pull speed [µm/s] | pH 4 | pH 7 vs pH 4 |
| 0.1 |  | 0.00114^(**)^ |
| 0.5 |  | 1.5E-10^(***)^ |
| 1 |  | 5E-23^(***)^ |
| 2 |  | 1.3E-29^(***)^ |
| 2.5 |  | 8E-32^(***)^ |
| 5 |  | 2E-23^(***)^ |
| 10 |  | 0.01103^(*)^ |

**Figure 3 – Adhesive work at different dwell times, pH, pulling speed. Hydrophilic tip**

Normality of data

a. pH 7, t1: mathematically none, graphically all around mean OK (outliers present)

b. pH 7, t2: same as t1

c. pH 4, t1: Mathematically, 2.5 – 10; graphically the others are also OK normally distributed.

d. pH 4, t2: Same as t1. Nearly all data have 2 outliers.

**1. Influence of increasing the pulling speed (Constant residence time)**

|  | pH 7 | | pH 4 | | |
| --- | --- | --- | --- | --- | --- |
|  | t1 | t2 | t1 | t2 |  |
| 0.1 |  |  |  |  |  |
| 0.5 | 0.70062 | 0.75724 | 4.06E-5^(***)^ | 0.15732 |  |
| 1 | 0.00959^(**)^ | 0.847 | 0.09501 | 0.45547 |  |
| 2 | 9.16E-4^(***)^ | 0.05851 | 0.25484 | 0.03514^(*)^ |  |
| 2.5 | 0.08342 | 0.79635 | 0.09346 | 0.85193 |  |
| 5 | 0.02801^(*)^ | 0.01744^(*)^ | 2.73E-6^(***)^ | 0.11437 |  |
| 10 | 0.3116 | 0.14622 | 0.01637^(*)^ | 0.66029 |  |

**2. Increasing the residence time (constant pulling speed)**

|  | pH 7 | pH 4 |
| --- | --- | --- |
|  | t1 vs t2 | t1 vs t2 |
| 0.1 | 0.39995 | 0.03527^(*)^ |
| 0.5 | 0.15846 | 0.16875 |
| 1 | 0.0023^(**)^ | 0.02361^(*)^ |
| 2 | 0.36469 | 1.2519E-5^(***)^ |
| 2.5 | 0.11335 | 5.668E-6^(***)^ |
| 5 | 0.16123 | 6.285E-7^(***)^ |
| 10 | 4E-6^(***)^ | 0.00535^(**)^ |

**3. Change of pH (constant Dwell time and pulling speed)**

|  | t1 | t2 |
| --- | --- | --- |
|  | pH 4 vs pH 7 | pH 4 vs pH 7 |
| 0.1 | 0.12876 | 0.44852 |
| 0.5 | 0.39979 | 0.08191 |
| 1 | 1E-5^(***)^ | 4.76E-5^(***)^ |
| 2 | 0.60163 | 0.01023^(*)^ |
| 2.5 | 0.89387 | 0.01166^(*)^ |
| 5 | 0.15471 | 0.13363 |
| 10 | 0.19676 | 0.01481^(*)^ |
